# Risk Factors of Fatigue in Idiopathic Parkinson's Disease in a Polish Population

**DOI:** 10.1155/2016/2835945

**Published:** 2016-01-26

**Authors:** Monika Gołąb-Janowska, Dariusz Kotlęga, Krzysztof Safranow, Agnieszka Meller, Anna Budzianowska, Krystyna Honczarenko

**Affiliations:** ^1^Chair and Clinic of Neurology, Pomeranian Medical University, Unii Lubelskiej 1, 71-252 Szczecin, Poland; ^2^Department of Biochemistry and Medical Chemistry, Pomeranian Medical University, Powstańców Wielkopolskich 72, 70-111 Szczecin, Poland; ^3^Medicinkliniken, Länssjukhuset Ryhov, 551 85 Jönköping, Sweden

## Abstract

*Introduction.* Fatigue syndrome is one of the nonmotor symptoms in Parkinson's disease (PD). The aim of the study was assessment of prevalence of fatigue syndrome in PD and answering the question what are the independent risk factors connected with intensity of fatigue in PD.* Methods*. 114 patients with idiopathic PD (mean age 62.2 + 10.8 years) were enrolled. The fatigue was assessed according to the Fatigue Severity Scale (FSS). We analyzed associations between fatigue and sex, age, education, duration and severity of the disease, everyday activity, intensity of the main symptoms, treatment, presence of dyskinesias and fluctuations, depression and excessive sleep during the day, and presence of pain and nycturia.* Results*. The fatigue syndrome was detected in 57.9% of patients. The score in the FSS was 1 to 7 points, 4.3 average. Greater fatigue intensity correlated with higher total daily levodopa equivalent dose. Patients with moderate depression had significantly greater fatigue.* Conclusions*. Fatigue syndrome affects 57.9% of patients with PD. Use of higher LED and presence of moderate depression are independent risk factors of greater intensity of fatigue.

## 1. Introduction

Nonmotor symptoms of Parkinson's disease (PD) are an important component of the clinical description [1, 2]. These symptoms, although not dominant, in a significant way affect the quality of life of both the patients and those caring for them [2, 3]. The most important groups of associated symptoms in PD comprise vegetative disorders and intellectual impairment including dementia and mental disorders, mostly depression. Fatigue, though less cumbersome than dementia or severe depression, is another common nonmotor symptom at PD affecting from 32 to 68% of the patients [4–6]. The interest in the problem of fatigue syndrome in PD has risen considerably in recent years. The first article assessing fatigue in PD was published in 1993 [7], and the first mention of the issue appeared in 1967 [8]. Fatigue is the single symptom most often cited as disabling by American PD patients who need to stop working [9]. The presence and severity of fatigue in PD are comparable to those reported by patients with multiple sclerosis, for whom the fatigue syndrome is one of the major problems [10].

There is no precise definition of excessive fatigue, due to the difficulty in distinguishing between common fatigue and fatigue being the manifestation of the disease. For clinical use fatigue is defined as a subjective feeling of lack of energy to start and maintain any activity, without any connection with depression or muscle weakness. Patients, describing the sensation, do not always use the word fatigue. They complain of drowsiness, difficulty in concentration, memory impairment (mental aspect), fatigability, weakness, and lack of energy to start an activity that previously caused no problems (physical aspect) [11]. Due to its heterogeneous nature, it is suggested that the term “fatigue syndrome” should be used [12]. The diagnosis of “primary” fatigue syndrome in the disease is difficult because of the close relationship between fatigue and other nonmotor symptoms of the disease.

Fatigue, which is one of the criteria for the diagnosis of major depression by DSM-IV [13], can appear as a consequence of sleep disorders and is related to apathy. However, it seems that fatigue, apathy, depression, and excessive sleepiness are independent symptoms occurring in PD [14, 15]. Studies into the subject showed a link between fatigue and other symptoms such as sleep disturbances, depression, anxiety, or sensory symptoms, on the basis of which the authors suggest that the distinction between primary fatigue associated with PD and the secondary fatigue is almost impossible [16]. It was only in Journal of Neurology in 2013 [17], to the knowledge of authors, was the first study published which separately described clinical determinants and psychosocial factors associated with different fatigue domains in primary and secondary fatigue in PD, underlining the importance of distinguishing primary and secondary fatigue in future PD studies and clinical practice.

Apathy in PD can be accompanied by depression and sometimes can be masked by it. It also may occur in PD patients without depression [15, 18]. This reduced sensitivity to emotional and physical stimuli, accompanied by lowered physical and mental activity and loss of interest, is considered a typical clinical symptom of dysfunction of the prefrontal cortex and basal ganglia, observed in PD [19, 20]. Apathy in PD is attributed to nigrostriatal dopaminergic loss in basal ganglia and is considered one of the core features of PD [18], occurring in up to 70% of PD patients [15, 21].

During a routine examination the level of fatigue in patients with PD cannot be objectively assessed. Among the many scales used in the assessment of its severity, the most common one is the Fatigue Severity Scale (FSS) [5, 22, 23].

The causes of fatigue in PD remain elusive. It is known that physical fatigue may be peripheral or central. Voluntary movements are generated by a sequence of events, and each of these events can be a place of the development of fatigue. These events include the entire central nervous system, stimulating the upper motor neuron in a way similar to a motivating factor and integrating sensory information, conduction along the pyramidal tract, activation of the lower motor neuron in the anterior ventral horn of the spinal cord, and conduction along the lower motor neuron and the neuromuscular junction [24]. Fatigue can also be caused by events that occur during exercise in the muscle [11, 24]. Central fatigue is defined as reduced power generation caused by events in/or proximal to the anterior horn. Peripheral fatigue is defined as a disorder in or out of a neuromuscular connection [11, 24]. It is unclear which of these two types of fatigue plays a more important role in PD. Also, it still has not been determined whether fatigue syndrome in PD is associated with nonspecific structural damage to the central nervous system as a result of the ongoing neurodegeneration or it is rather a consequence of the selective destruction of neurotransmitter pathways [25]. Fatigue is present in many neurological diseases and mental and somatic disorders, so it seems improper to assume that it occurs as a result of damage in a particular region of the brain. However, there is a hypothesis which appears to connect all the fatigue-accompanied diseases. The hypothesis is based on the assumption that fatigue is associated with impaired motivation, which may result from the impact of disorders of the nigrostriatal pathway. It is supported by the fact that SPECT examination of PD patients revealed a considerable reduction of perfusion in the frontal lobes [26], compared to patients without fatigue [27].

The aim of this study was to assess the prevalence of fatigue in Parkinson's disease and answer the following question: what are the independent factors associated with the severity of fatigue in PD?

We analyzed the relationship between sex, age, education, disease duration, severity and stage of the disease, activity of daily life, severity of axial symptoms, the presence of dyskinesia and fluctuations, treatment applied, and the relationship between concomitant depression and excessive daytime sleepiness. We also studied the connection between fatigue and the presence of pain and nocturia, which may affect the perception of fatigue by patients.

## 2. Methods

### 2.1. Participants

The study included 114 out of the 158 patients with a diagnosis of idiopathic Parkinson's disease established according to the current UK PDSBB (Parkinson's Disease Society Brain Bank) [28] criteria and treated in the Outpatient Extrapyramidal Disease and Memory Disorders Clinic at the Department of Neurology of PUM in Szczecin, Poland.

The study group consisted of 70 (61.4%) men and 44 (38.6%) women aged between 25 and 80, with the average age of 62.2. Eighteen patients had primary education and 30 had vocational, 40 had secondary, and 26 had higher education.

*Exclusion Criteria*. Exclusion criteria were as follows:

- Lack of consent to participate in the study.
- Comorbid dementia (score below 24 points in Mini Mental Status Examination) [29].
- Coexisting systemic disease that can cause fatigue (anemia, renal failure, cardiac failure, hypothyroidism, and uncontrolled diabetes mellitus).

The history was taken by means of a unique form sheet, which included data on age of onset, the beginning, duration, course of the disease, the prevalence of complications, tolerability and efficacy of treatment, comorbidity of other diseases, and the use of chronic medication. A total daily levodopa equivalent dose (LED) was calculated based on previous reports with LED: (regular levodopa dose × 1) + (levodopa controlled-release dose × 0.75) + (pramipexole dose × 0.67) + (ropinirole dose × 16.67) + (rotigotine × 16.67) + (pergolide dose and cabergoline dose × 0.67) + (bromocriptine dose × 10) + ([regular levodopa dose + levodopa controlled-release dose × 0.75] × 0.25) if taking tolcapone or entacapone [30].

### 2.2. Procedure

The neurological examination performed evaluated the following features: severity of the disease and the presence of side effects of treatment by means of the Unified Parkinson's Disease Rating Scale (UPDRS) [31], the stage of the disease (the Hoehn and Yahr Stage scale) [8], and the activity of daily living (the Schwab and England scale) [32].

For the diagnosis of depression the Beck Depression Inventory (BDI) was used, in the version proposed by Puzynski and Wciorka [33, 34]. Excessive daytime sleepiness was assessed by means of the Epworth Sleepiness Scale (ESS) [35]. The Brief Pain Inventory-Short Form [36] was used to evaluate pain. In the analysis, though, the aspect of absence or presence of pain was included.

In order to assess the fatigue the clinic's own translation of the FSS was used. The scale is commonly used in neurological diseases, including PD [5, 22, 23]. The scale was translated from the English version (under the current rules) by a team comprising a translator and a neurologist. The resulting translation was then compared with the original and the small differences in the translation were discussed within the team. Eventually, an independent bilingual person made a back translation followed by subsequent correction of any inaccuracies in the Polish version. The scale consists of nine items assessing the impact of fatigue on daily activity, motivation, work, and family life. Individual items closely reflecting the condition of the patient over the last week are evaluated in a 7-point (1–7) estimate scale. Low value (e.g., 1) shows a strong disagreement with the statement, while a high one (e.g., 7) shows strong agreement. The result of the test is the sum of individual items of the scale of points divided by the number of items (this is the average score of all items). It was decided that fatigue occurs when the result is higher than or equal to 4 points.

The study was approved by the Pomeranian Medical University Commission of Ethics, Resolution number KB-0012/07/10 of 21 Jan. 2011.

### 2.3. Statistical Analyses

Most measurable variables showed distributions significantly different from a normal distribution (Shapiro-Wilk test, *p* < 0.05), because in their analysis nonparametric statistical tests were used: in the Mann-Whitney test for comparisons between groups and the Spearman rank correlation coefficient to assess the strength of correlation between the variables. Nominal variables were compared by means of chi-square test or exact Fisher's test. Multivariate analysis was performed based on the general linear model (GLM). As the threshold for statistical significance was assumed *p* < 0.05.

## 3. Results

The duration ranged from 0.5 years to 24 years, on average 12.3 years. In the UPDRS (carried out in phase on) patients received from 10 to 81 points, on average 37.3 points; in part III of the UPDRS (also for phase on) they received from 6 to 36 points, on average 18.2 points. In the Hoehn and Yahr scale patients received from 1 to 4 points, on average 2.3 points. In the Schwab and England scale they received from 40 to 100%, on average 77%. Slowdown on the scale of 0 to 4 was as follows: 2 patient rated 0 (1.7%), 30 rated 1 (26.3%), 54 rated 2 (47.4%), and 28 rated 3 (24.6%); stiffness was rated 0 in 10 patients (8.8%), 1 in 34 (29.8%), 2 in 50 (43.9%), and 3 in 20 (17.5%); tremor was rated 0 in 24 (21.1%), 1 in 30 (26.3%), 2 in 36 (31.6%), 3 in 22 (19.3%), and 4 in 2 patients (1.7%); basic reflexes disorders were rated 0 in 10 patients (8.8%), 1 in 66 (57.9%), 2 in 36 (31.6%), and 3 in 2 (1.7%). Dyskinesia occurred in 36 patients (31.6%), and fluctuations occurred in 46 (40.4%).

LED was of 200 to 2000 mg, 504.4 mg. 42 patients (36.8%) complained of pain. Nocturia was reported by 52 patients (45.6%). 70 patients (61.4%) were not diagnosed with depression; mild depression was present in 38 (33.3%) and moderate in 6 patients (5.3%); none of the patients suffered from severe depression. Excessive daytime sleepiness was found in 14 patients (12.3%), and pathological sleepiness was found in another six (5.3%). Fatigue syndrome was diagnosed in 66 patients (57.9%). On the FFS patients received from 1 to 7 points, with the median of 4.3 points. The mean and standard deviation of the results obtained in different positions on the FFS were shown in Table 1.

We found a statistically significant correlation between the increased severity of fatigue syndrome and the higher LED (*R*_*s*_ = 0.30, *p* = 0.025). Patients with moderate depression felt significantly more intense fatigue than patients without depression (6.8 ± 0.2 versus 4.0 ± 1.6 on FSS, *p* = 0.0002). The FFS value did not correlate with the patients' age (*R*_*s*_ = 0.12, *p* = 0.39) or the duration of illness (*R*_*s*_ = 0.18, *p* = 0.18). Comparing the group of patients with FFS ≥ 4 versus FFS < 4 we did not identify any relationship between the presence of fatigue syndrome and sex, age, education, duration of the disease, severity of the disease, stage of the disease, activity of daily life, severity of axial symptoms (slowing down, tremor, rigidity, and basic reflexes disorders), presence of complications during the treatment (dyskinesia, fluctuation), excessive daytime sleepiness, the presence of pain, and the occurrence of nocturia (Table 2).

Despite failure to meet certain assumptions of the general linear model (far below from the normal distribution schedule of LED and duration of PD), multivariate analysis was performed where the dependent variable was the value of FFS and the independent variables were age, sex, duration of PD, LED, and the presence of moderate depression. The adequacy of such a model was supported by the fact that the distribution of residuals (the difference between the observed and predicted FSS values) did not deviate significantly from the normal distribution (*p* = 0.23, Shapiro-Wilk test). Independent factors associated with more severe fatigue considering age, sex, and PD duration were as follows: higher LED (by 0.16 points on the FSS for each 100 mg/d LED, 95% confidence interval 0.05–0.27 points, *p* = 0.0047) and the presence of moderate depression (2.9 points on the FSS, 95% confidence interval 0.98–4.82 points, *p* = 0.0039). There is no relationship between the FSS score and age and sex and duration of the disease.

## 4. Discussion

The first studies into the assessment of fatigue in PD were published in 1993 [7], but 26 years earlier Hoehn and Yahr noted that fatigue is one of the symptoms of PD, present in approximately 3% of the patients [8]. As research shows, taking into account population differences, differences in the definition of fatigue, and the use of different measurement instruments, fatigue can affect from 3 to 68% of PD patients [6, 14, 16, 37–39]. The first US study found that fatigue affected 58% of patients with PD, and in 33% of patients it was (including movement disorder) one of the most function limiting symptoms [7]. 68% of patients assessed the fatigue associated with PD as qualitatively different from the fatigue which they felt before the onset of the disease. The average score of the severity of fatigue measured on FSS was similar to the results obtained from patients with MS. Fatigue syndrome was linked to depression but was not associated with the severity of movement disorders [7]. In the Dutch study [40] evaluating fatigue in the population of Dutch PD patients a higher incidence of fatigue in PD patients was observed in comparison with the control group. The authors did not assess the severity of fatigue. Only the subsequent Dutch study [37] showed that fatigue syndrome was present in up to 43% of patients with Parkinson's disease without accompanying depression. In approximately half of the patients fatigue syndrome preceded the appearance of motor symptoms of the disease. 15% of the examined group rated fatigue as the most disruptive symptom of the disease, and more than half claimed that it was stronger than the other symptoms. Fatigue syndrome was diagnosed in 44.2% of patients with Parkinson's disease, in a large (233 patients) study of the Norwegian population, which was in sharp contrast to the 18% incidence in healthy, age-comparable control group. Fatigue remained in a significant relation with depression [41], but not with disease severity. It is interesting and important that fatigue syndrome occurred in patients both with and without concomitant depression, excessive daytime sleepiness, and dementia [39]. Shulman and partners [16] evaluated a selected group of American patients with PD from Baltimore and found out the presence of fatigue in 44% of them. Fatigue has been shown to correlate with anxiety and decreased activity of daily living. No association with sex, age, presence of depression or sleep disorders was found. Another study conducted in southeastern America helped identify fatigue syndrome in 40% of patients with Parkinson's disease [42]. In the Japanese study, Abe and colleagues [27] compared the fatigue frequency in 26 patients with Parkinson's disease without coexisting dementia to 26 healthy subjects who comprised a comparable control group in terms of sex and age. They proved a more frequent fatigue syndrome in patients with PD. Fatigue was found in one-third of patients with early Parkinson's disease involved in a clinical trial ELLDOPA [43]. In 128 out of 349 examined, fatigue syndrome was already present at the time the patients were qualified for the test, which, according to the authors, proves that fatigue is a common early symptom of PD.

In our own material fatigue syndrome was found in 57.9% of the patients, which, although relatively frequent, is not in contradiction with the results obtained by other authors.

The relationship between fatigue and age and sex was evaluated in the already cited study by Shulman et al. [16]. The authors found no relationship between fatigue syndrome and either sex or age. Also, in the studies by Herlofson and Larsen [5] and Martinez-Martin et al. [6], in which the incidence of fatigue was estimated to be 67.6%, there was no association between the severity of fatigue syndrome and age, sex, disease duration, and duration of treatment, which is consistent with the results of our study. In the study by Martinez-Martin et al. [6] a tendency to higher results obtained in the Hoehn and Yahr scale was only observed in patients with fatigue.

The study by Elbers et al. [44] showed an increase in movement disorders assessed using the UPDRS Part III and the stage of the disease remained in a significant relationship with the severity of fatigue, which is consistent with the results of two other studies [4, 45]. Herlofson and Larsen [5] demonstrated the relationship of fatigue with a greater severity and stage of the disease as well as increased severity of postural disorders unrelated to the intensification of other movement disorders (slowing, rigidity, and tremor). Many authors [10, 27, 39] showed no relationship between the severity of fatigue and increased movement disorders related to the severity of the disease, its stage, everyday activity, and the severity of axial symptoms of Parkinson's disease (slowing rigidity, tremor, and basic reflexes disorders), which is consistent with the results obtained in our study.

As previously mentioned, it is unclear whether fatigue syndrome in Parkinson's disease is associated with nonspecific structural damage to the central nervous system as a result of the ongoing neurodegeneration or rather with a consequence of the selective destruction of neurotransmitter pathways [25]. The neurotransmitter dysfunction hypothesis seems to be supported by the fact that after the administration of pergolide (a selective agonist of D1 and D2) reduction of fatigue as well as, visible on the PET scan image, damage to the basal ganglia was observed in MS patients suffering from fatigue [46]. This hypothesis would be also consistent with the previously described fatigue susceptibility to L-dopa treatment. Objectively measured muscle strength increases after the administration of L-dopa preparations, regardless of the results obtained in the Multidimensional Fatigue Inventory (MFI). A bilateral stimulation of the subthalamic nuclei provides a similar effect [47, 48].

Studies by Lou et al. [47] showed that L-dopa reduces physical fatigue in Parkinson's disease, which is a well-known effect of L-dopa in the central dopaminergic system. The authors suggest that a central fatigue may play a more important role in patients with Parkinson's disease. The 42-week-long ELLDOPA study [43], in which fatigue was assessed in patients with previously untreated early Parkinson's disease, proved that fatigue intensified significantly in patients who were not treated with L-dopa.

Also, the results of earlier research suggested that dopaminergic treatment can improve at least some aspects of fatigue [46]. The study by Herlofson and Larsen [5] showed a weak positive relationship between fatigue and the daily dose of L-dopa, the duration of dopaminergic treatment, and severity of the disease. The follow-up multivariate analysis, however, did not confirm any significant relationship between fatigue syndrome and the variables analyzed in the test.

The existing publications do not confirm the connection between fatigue syndrome and duration of treatment and type of medications administered (except amantadine in the study of Martinez-Martin et al. [6], as the percentage of patients with fatigue syndrome treated with amantadine was significantly lower).

In our material we discovered a significant statistical correlation between the severity of fatigue and a higher LED, which is consistent with data from the literature [43, 46, 47] showing that fatigue as a symptom is sensitive to dopamine. The LED proved to be an independent factor associated with the severity of fatigue in multivariate analysis taking into account age, sex, and duration of PD.

Fatigue is closely related to other nonmotor symptoms of PD. Studies into the connections between fatigue and other nonmotor symptoms such as sleep disorders, depression, anxiety, and sensory symptoms have shown that at least one symptom occurs in 88% of the patients with PD, two or more occur in 59%, three or more occur in 39%, four or more occur in 23%, and more than five occur in 11%. A larger number of coexisting symptoms are associated with the increasing severity of motor deficits [16]. Shulman and colleagues [16] suggest that it is virtually impossible to distinguish between “primary fatigue” associated with Parkinson's disease and “secondary fatigue” appearing in patients with PD in the course of conditions such as depression or daytime sleepiness. There are also studies showing fatigue as an independent symptom of PD, not associated with excessive daytime sleepiness or night sleep disorders [4, 49]. Similarly, although depression disorders are common in Parkinson's disease and fatigue is one of the criteria for the diagnosis of major depression according to DSM-IV, it is believed that both fatigue syndrome and depression are independent symptoms occurring in PD [4]. Chaudhuri and Behan [11] postulated that fatigue in neurological diseases may be caused by broken connections between the prefrontal cortex and the thalamus. Damage to the basal ganglia may affect limbic integration for voluntary actions controlled by the cortex.

Several epidemiological studies looked into the relationship between fatigue and depression in patients with PD. Using the multivariate logistic regression model, the presence of fatigue syndrome was assessed in both patients with and without depression [39]. Fatigue was observed in 43.5% of the patients with no coexisting depression, dementia, or sleep disorders. The authors believe that fatigue syndrome is a common problem that may appear regardless of depression and sleep disorders.

The natural course of fatigue syndrome in PD is uncertain due to the fact that merely a few long-term observations are recorded in literature on the subject. Alves et al. [4] monitored 233 PD patients for 8 years, assessing fatigue first at the start of the study, then after 4 years, and eventually after 8 years. They noted an increased incidence of fatigue, which affected, respectively, 35.6%, 42.9%, and 56% of the patients. In a separately assessed group of PD patients with coexisting depression and excessive daytime sleepiness fatigue syndrome was reported in 32% at the beginning of the study and in 39% at the end. Fatigue in the majority of patients afflicted with fatigue syndrome had a permanent character; in 44% it was intermittent. Fatigue syndrome was found to be associated with the stage of the disease, depression, and excessive daytime sleepiness, even in patients without depression. These results are in contradiction with the results obtained in a small observational study, which showed that in the majority of the patients with fatigue present at the start of the study fatigue remained present in the phase of the examination, whereas the patients who did not show fatigue at the first evaluation, were unlikely to develop it later [7]. Fatigue showed a tendency to increase in time.

In our study, we have shown the relationship between more severe fatigue and the presence of moderate depression; we have not found a higher incidence of fatigue syndrome in patients with excessive daytime sleepiness.

We assumed that fatigue in PD may be associated with pain and disrupting night sleep nocturia. However, we have found no relationship between the frequency fatigue and pain occurred, which is consistent with the results obtained by Herlofson and Larsen [5]. Like Herlofson and Larsen [5], we have found no increase in the incidence of fatigue syndrome in patients suffering from nocturia.

## 5. Conclusions

Fatigue is a frequent symptom in patients with idiopathic Parkinson's disease, affecting more than 50% of these patients. The use of higher doses of L-dopa and the presence of moderate depression are independent factors associated with a higher severity of fatigue.

## Figures and Tables

**Table 1:** Fatigue Severity Scale (FSS).

		PD
		(*n* = 114)
FSS 1	My motivation is lower when I am fatigued	4.9 (2.1)
FSS 2	Exercise brings on my fatigued	5.3 (1.9)
FSS 3	I am easily fatigued	4.5 (2.0)
FSS 4	Fatigue interferes with my physical functioning	4.3 (2.1)
FSS 5	Fatigue causes frequent problems for me	4.0 (2.1)
FSS 6	My fatigue prevents sustained physical functioning	4.2 (2.1)
FSS 7	Fatigue interferes with carrying out certain duties and responsibilities	4.1 (2.1)
FSS 8	Fatigue is among my 3 disabling symptoms	3.6 (2.1)
FSS 9	Fatigue interferes with my work, family or social life	3.8 (2.1)
FSS	Total score	4.3 (1.7)

**Table 2:** Descriptive data for patients with PD and all patients and for patients with (FSS ≥ 4) and without (FSS < 4) fatigue separately.

	All PD patients	Fatigued patients	Nonfatigued patients	*p* ^*∗*^
	(*n* = 114)	(*n* = 66)	(*n* = 48)	
Gender, *n* (%) female	44 (38.6%)	26 (39.4%)	18 (37.5%)	1.0

Age, years	62.2 (10.8)	62.6 (12.2)	61.6 (8.9)	0.58
	61 [25–80]	61 [25–80]	61 [46–78]	

Education, *n* (%):				
Primary	18 (15.8%)	10 (15.2%)	8 (16.7%)	0.98
Vocational	30 (26.3%)	18 (27.3%)	12 (25.0%)	
Secondary	40 (35.1%)	22 (33.3%)	18 (37.5%)	
Higher	26 (22.8%)	16 (24.2%)	10 (20.8%)	

Disease duration, years	12.3 (16.9)	14.7 (19.9)	9.1 (11.3)	0.23
	6 [0.5–72]	7 [0.5–72]	5.5 [0.5–54]	

UPDRS, score	37.3 (18.9)	39.6 (17.6)	34.2 (20.5)	0.18
	32 [10–81]	37 [17–79]	28.5 [10–81]	

UPDRS Part III, score	18.2 (8.6)	18.5 (7.7)	17.7 (9.9)	0.47
	16 [6–36]	17 [7–36]	14.5 [6–36]	

Hoehn and Yahr, stage	2.3 (0.8)	2.5 (0.8)	2.1 (0.8)	0.13
	2.5 [1–4]	2.5 [1–4]	2.0 [1–4]	

Schwab and Englanda, score	77 (12)	75 (11)	79 (14)	0.12
	80 [40–100]	80 [50–90]	80 [40–100]	

Rigidity score	1.7 (0.9)	1.6 (0.9)	1.8 (0.8)	0.81
	2 [0–3]	2 [0–3]	2 [0–3]	

Tremor score	1.5 (1.1)	1.4 (1.0)	1.7 (1.2)	0.40
	2 [0–4]	2 [0–3]	1.5 [0–4]	

Postural abnormalities score	1.3 (0.6)	1.4 (0.6)	1.1 (0.7)	0.09
	1 [0–3]	1 [0–3]	1 [0–2]	

Akinesia score	1.9 (0.8)	2.0 (0.7)	1.9 (0.9)	0.88
	2 [0–3]	2 [1–3]	2 [0–3]	

Dyskinesias, *n* (%)	36 (31.6%)	22 (33.3%)	14 (29.2%)	0.78

Fluctuations, *n* (%)	46 (40.4%)	30 (33.3%)	36 (31.6%)	0.42

Treatment duration, years	5.2 (5.8)	7.0 (5.9)	5.1 (5.3)	0.19
	5 [0–23]	6 [0–23]	4 [0–18]	

LDE, mg/day	504.4 (393.5)	589.4	387.5	0.10
	500 [0–2000]	(428.6)	(311.1)	

BDI, *n* (%):				
Normal	70 (61.4%)	38 (57.6%)	32 (66.7%)	0.31
Mild depression	38 (33.3%)	22 (33.3%)	16 (33.3%)	
Moderate depression	6 (5.3%)	6 (9.1%)	0 (0%)	
Severe depression	0 (0%)	0 (0%)	0 (0%)	

ESS, *n* (%):				
Normal	94 (82.5%)	54 (81.2%)	40 (83.3%)	0.53
Mild-to-moderate sleep apnea	14 (12.3%)	10 (15.2%)	4 (8.3%)	
Severe sleep apnea, ESS > 15	6 (5.3%)	2 (3.0%)	4 (8.3%)	

Presence of pain, *n* (%)	42 (36.8%)	26 (39.4%)	16 (33.3%)	0.78

Presence of nycturia, *n* (%)	52 (45.6%)	26 (39.4%)	26 (54.2%)	0.29

The table presents standard deviation (SD) and median (min–max) for measurable variables or numbers, and proportions for nominal variables.

^*∗*^Significance of the difference between the patients' groups with FSS ≥ 4 and FSS < 4; Mann-Whitney *U* test for measurable and rank variables and Fisher's exact test for nominal variables.
